# Susceptibility Genes and Chromosomal Regions Associated With Non-Syndromic Familial Non-Medullary Thyroid Carcinoma: Some Pathogenetic and Diagnostic Keys

**DOI:** 10.3389/fendo.2022.829103

**Published:** 2022-02-28

**Authors:** María Sánchez-Ares, Soledad Cameselle-García, Ihab Abdulkader-Nallib, Gemma Rodríguez-Carnero, Carolina Beiras-Sarasquete, José Antonio Puñal-Rodríguez, José Manuel Cameselle-Teijeiro

**Affiliations:** ^1^ Department of Pathology, Clinical University Hospital of Santiago de Compostela, Health Research Institute of Santiago de Compostela, Galician Healthcare Service (SERGAS), Santiago de Compostela, Spain; ^2^ Department of Medical Oncology, University Hospital Complex of Ourense, Galician Healthcare Service (SERGAS), Ourense, Spain; ^3^ School of Medicine, University of Santiago de Compostela, Santiago de Compostela, Spain; ^4^ Department of Endocrinology and Nutrition, Clinical University Hospital of Santiago de Compostela, Galician Healthcare Service (SERGAS), Santiago de Compostela, Spain; ^5^ Department of Surgery, Clinical University Hospital of Santiago de Compostela, Galician Healthcare Service (SERGAS), Santiago de Compostela, Spain

**Keywords:** familial non-medullary thyroid carcinoma, familial thyroid cancer, papillary thyroid carcinoma, susceptibility genes, cancer risk, cancer diagnosis

## Abstract

Thyroid cancer is the malignant tumor that is increasing most rapidly in the world, mainly at the expense of sporadic papillary thyroid carcinoma. The somatic alterations involved in the pathogenesis of sporadic follicular cell derived tumors are well recognized, while the predisposing alterations implicated in hereditary follicular tumors are less well known. Since the genetic background of syndromic familial non-medullary carcinoma has been well established, here we review the pathogenesis of non-syndromic familial non-medullary carcinoma emphasizing those aspects that may be useful in clinical and pathological diagnosis. Non-syndromic familial non-medullary carcinoma has a complex and heterogeneous genetic basis involving several genes and loci with a monogenic or polygenic inheritance model. Most cases are papillary thyroid carcinoma (classic and follicular variant), usually accompanied by benign thyroid nodules (follicular thyroid adenoma and/or multinodular goiter). The possible diagnostic and prognostic usefulness of the changes in the expression and/or translocation of various proteins secondary to several mutations reported in this setting requires further confirmation. Given that non-syndromic familial non-medullary carcinoma and sporadic non-medullary thyroid carcinoma share the same morphology and somatic mutations, the same targeted therapies could be used at present, if necessary, until more specific targeted treatments become available.

## Introduction

Thyroid cancer (TC) is the most increasing malignancy in the world, mainly due to the diagnosis of papillary thyroid carcinoma (PTC), which is the most common histological type of TC (1). While *RET* gene mutations play a fundamental role in the development of C cell-derived thyroid carcinomas including the familial forms of medullary thyroid carcinoma (2), the genetic-molecular profile of follicular cell-derived TC is more heterogeneous. Hereditary follicular cell TC is commonly referred to as familial non-medullary TC (FNMTC) and its genetic basis remains to be established (3, 4).

Most authors agree that the clinical diagnosis of FNMTC is based on the evidence of papillary thyroid carcinoma (PTC) in two or more first-degree relatives, or on the finding of multinodular goiter (MNG) in at least three first- or second-degree relatives of a PTC patient, always in the absence of a history of ionizing radiation exposure (3, 4). FNMTC is generally classified into two large groups: syndromic familial non-medullary thyroid cancer (S-FNMTC) when the TC is associated with syndromes that have extrathyroid manifestations, and non-syndromic familial non-medullary TC (NS-FNMTC) (3, 4). S-FNMTC is generally secondary to germline mutations in the *APC*, *PTEN*, *DICER1*, *PRKAR1A*, or *WRN* genes (3, 4). The characteristics of S-FNMTC are better defined, which means that sometimes, from the histopathological and/or immunohistochemical features of the thyroid tumors, the existence of a *PTEN*-hamartoma tumor syndrome or a familial adenomatous polyposis can be suggested; this condition, however, must be genetically confirmed from the patient’s blood samples (4, 5). In contrast, in families with predominance of TC (NS-FNMTC) the clinicopathological profile is less well defined and its genetic basis remains unclear.

**Figure 1:**
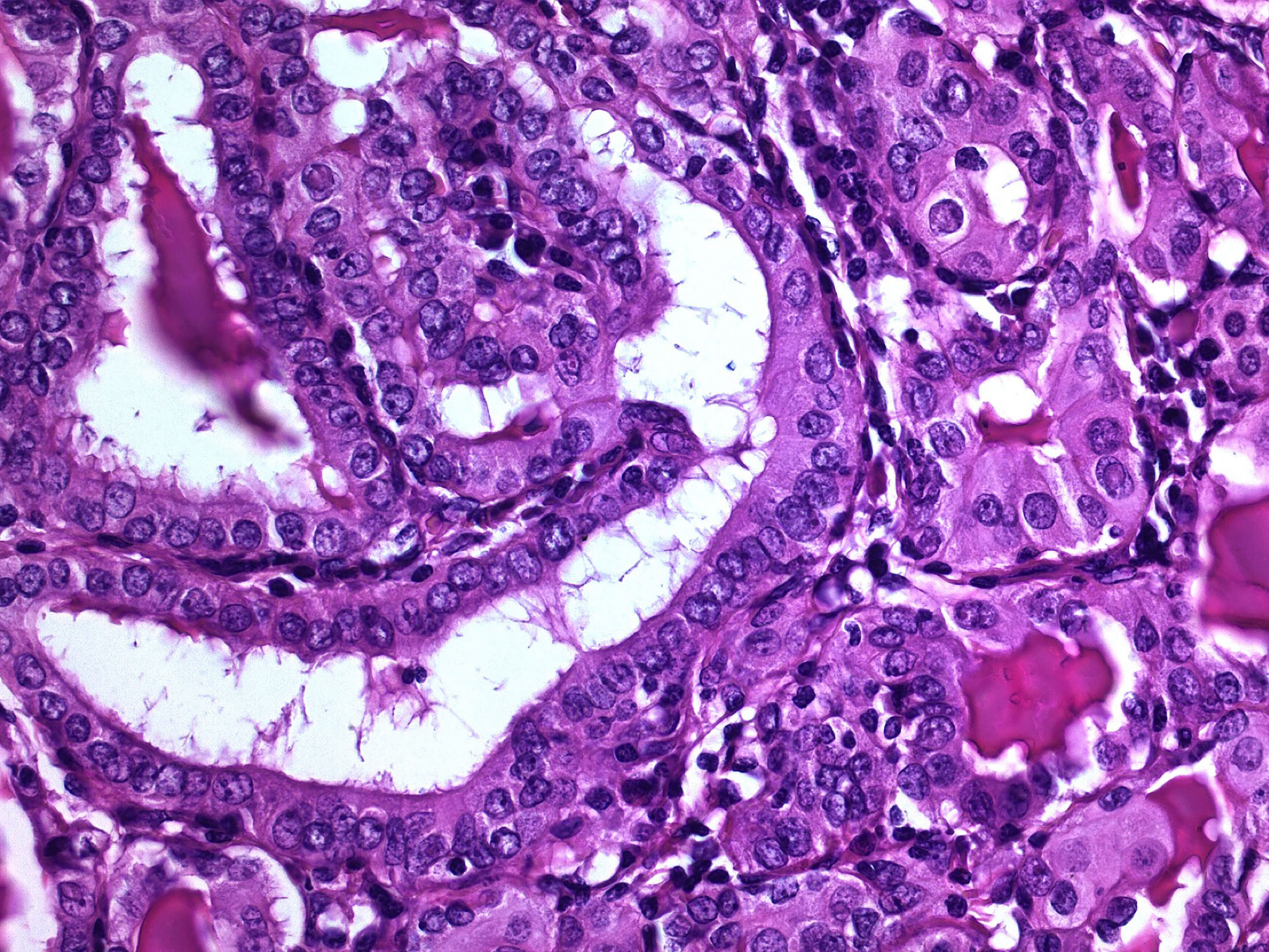
Non-syndromic familial papillary thyroid carcinoma. This tumor occurred in a patient with two first-degree relatives having non-medullary thyroid carcinoma, in the absence of a history of ionizing radiation exposure (hematoxylin-eosin, 400X). Typically, these familial thyroid tumors are not morphologically different from their sporadic counterpart.

This lack of knowledge concerning the biology of NS-FNMTC could be the result of the absence of a consensus in the clinical definition of NS-FNMTC, the lack of typical tumor histopathological features and/or the number of putative NS-FNMTC susceptibility genes that have been described. In fact, it has been calculated that when the FNMTC definition is based on the presence of two affected members, the probability of a family origin is 47%, while when it is based on a minimum of 3 first-degree relatives, the probability of being related rises to more than 95% (6–8). Clinical studies show that NS-FNMTC shows more extrathyroidal extension, multifocality, combination with both benign thyroid nodules and Hashimoto disease, and a younger age at onset (3, 4, 9). In contrast, in most articles describing susceptibility genes for NS-FNMTC, however, the histopathological characteristics of TC are usually not very precise (**Figure 1**). In this work, we review the main NS-FNMTC susceptibility genes reported (**Tables 1** and **2**), including the main thyroid tumor characteristics, while also emphasizing those aspects that may be useful in clinical and pathological diagnosis. Additional therapeutic considerations are also discussed. Both the susceptibility genes and chromosomal regions associated with NS-FNMTC are listed below following the order of their chromosomal location.

**Table 1:** Susceptibility genes associated with non-syndromic familial non-medullary thyroid tumors.

Location	Gene (Full name)	Gene (Symbol)	Thyroid findings	References
1p13.2	WD repeat domain 77	*WDR77*	PTC	(10)
1q41	BRO1 domain and CAAX motif containing	*BROX*	PTC	(11)
1p36.31	Pleckstrin homology and RhoGEF domain containing G5	*PLEKHG5*	PTC	(12)
4q21.21	Annexin A3	*ANXA3*	PTC, HCC	(12)
6p21.33	Suppressor APC domain containing 1	*SAPCD1*	PTC	(12)
7q31.33	Protection of telomeres 1	*POT1*	MNG, HCA, PTC, HCC	(13–16)
9q22.33	Forkhead box E1	*FOXE1/TTF2*	PTC, FTC	(17–19)
10q25.3	Hyaluronan binding protein 2	*HABP2*	FTA, PTC	(20, 21)
12q14.2	SLIT-ROBO Rho GTPase activating protein 1	*SRGAP1*	PTC	(22)
12q22	Netrin 4	*NTN4*	PTC, HCC	(12)
14q11.2	Chromosome 14 open reading frame 93	*C14orf93/RTFC*	PTC	(23)
14q13.3	NK2 homeobox 1	*NKX2-1/TTF1*	MNG, PTC	(12, 24, 25)
14q32.13	Serpin family A member 1	*SERPINA1*	PTC	(12)
15q21.1	Dual oxidase 2	*DUOX2*	PTC	(26)
15q23	Mitogen-activated protein kinase kinase 5	*MAP2K5*	PTC	(27)
16p13.3	Serine/arginine repetitive matrix 2	*SRRM2*	PTC	(28)
17p13.2	Purinergic receptor P2X 5	*P2RX5*	PTC	(12)
17q21.2	FKBP prolyl isomerase 10	*FKBP10*	PTC	(12)
19p13.11	NADH:ubiquinone oxidoreductase subunit A13	*NDUFA13*	Oncocytic nodules, PTC (Hürthle cell variant)	(29)
19p13.2	Translocase of inner mitochondrial membrane 44	*TIMM44*	Oncocytic nodules, FTA, PTC (also including the oncocytic cell variant), HCC	(30)
19q13.33	NOP53 ribosome biogenesis factor	*NOP53*	MNG, PTC, HCC	(31)
20p12.3	Phospholipase C beta 1	*PLCB1*	MNG, PTC	(32–34)
22q12.1	Checkpoint kinase 2	*CHEK2*	PTC	(35–38)

PTC, papillary thyroid carcinoma; HCC, oncocytic (Hürthle cell) carcinoma; MNG, multinodular goiter; HCA, oncocytic (Hürthle cell) adenoma; FTA, follicular thyroid adenoma.

**Table 2:** Chromosomal loci associated with non-syndromic familial non-medullary thyroid tumors.

Location	Proposed full name for the unknown gene	Proposed symbol	Thyroid findings	References
1q21	Familial PTC with papillary renal neoplasia	*fPTC/PRN*	MNG, PTC	(39, 40)
2q21	Non-medullary thyroid carcinoma 3	*NMTC3*	PTC	(41–43)
8p23.1-p22	Familial thyroid epithelial neoplasia	*FTEN*	PTC	(44)
8q24.22	Papillary thyroid carcinoma susceptibility candidate 1	*PTCSC1*	MNG, PTC	(45)
14q^#^	Multinodular goiter 1	*MNG1*	MNG, PTC, FTC	(46)
14q.13.3	Papillary thyroid carcinoma susceptibility candidate 3	*PTCSC3*	PTC	(47)
19p13.2^$^	Thyroid tumor with cell oxyphilia	*TCO*	MNG, PTC (including the oncocytic variant), HCC, FTA, oncocytic nodules	(48, 49)

MNG, multinodular goiter; PTC, papillary thyroid carcinoma; FTC, follicular thyroid carcinoma; HCC, oncocytic (Hürthle cell) carcinoma; FTA, follicular thyroid adenoma.

^#^Some of these cases reported in children with MNG and follicular thyroid carcinomas could really be secondary to a germline mutation of the DICER1 gene located at 14q32.13 (50–52), or could be due to germinal mutations of the NKX2-1 gene located at 14q13.3 (25).

^$^At least some of these cases are secondary to germline mutations of the MYO1F (53) or TIMM44 (30) genes, both located at 19p13.2.

## Susceptibility Genes Associated With NS-FNMTC

### WD Repeat Domain 77 (*WDR77*)

The gene *WDR77* (WD repeat domain 77) has been recently reported to predispose to familial PTC in two unrelated Chinese families (10). By means of whole-exome sequencing (WES) the authors found two germline loss-of-function variants occurring within a 28 bp fragment of *WDR77*. A heterozygous missense mutation (p.R198H) in *WDR77* exon 6 was identified in one family of three affected siblings, and a heterozygous splice-site mutation (c.619+1G>C) at the 5’ end of intron 6 was present in three affected members from the other family. The two variants were validated in affected family members (4 women and 2 men) by Sanger sequencing (Ssq), indicating an autosomal dominant inheritance pattern of PTC associated with mutations in *WDR77*. The age of the patients at the diagnosis of PTC (including papillary and follicular variants) ranged from 33 to 57 years (mean diagnosis age 44.6 yrs.) and small thyroid nodules were detected in another member of the affected families; in addition to PTC (43 yrs.), an invasive ductal breast cancer (36 yrs.) had been diagnosed in one of the patients with the c.619+1G>C mutation. Somatic *BRAF* ^V600E^ mutation was detected in one PTC case, but neither somatic mutations in *KRAS*, *NRAS*, *HRAS* and *TERT* (c.-124C>T and c.-146C>T) nor germline mutations in *PTEN*, *APC*, *DICER1*, *WRN*, *PRKAR1A*, *STK11*, *SPGAP1*, *NKX2.1*, *FOXE1*, *HABP2*, and *CHEK2* were detected in these PTC patients (10).

*WDR77* encodes a core member of a transmethylase complex formed with the protein arginine methyltransferase 5 (*PRMT5*) that is responsible for histone H4 arginine 3 dimethylation (H4R3me2) in mammals. WDR77 protein is also known as methylosome protein 50 (*MEP50*) and androgen receptor cofactor p44 (*p44*) (54). Functional studies of *WDR77* variants in PTC patients showed that these variants impair formation of the complex WDR77-PRMT5, resulting in reduced H4R3me2 in patients, and knockdown of *WDR77* resulted in increased growth of thyroid cancer cells (10). The functional activity of this protein is related to its cytoplasmic location; for example, the WDR77 protein is essential for cellular proliferation when it localizes in the cytoplasm of epithelial cells at the early stage of prostate development (55). In the adult prostate tissue, this protein is transported into the nucleus and functions as a co-regulator of the androgen receptor to promote cellular differentiation. This process is reversed in prostate tumorigenesis, so that WDR77 protein is translocated from the nucleus into the cytoplasm, promoting proliferation of prostate cancer cells (54, 55). Interestingly, in the study by Zhao et al. (10) the cytoplasmic and nuclear immunopositivity for WDR77 of PTC cells contrasts with the higher intensity of nuclear staining of normal follicular cells adjacent to the tumor. Additional studies should confirm the role of immunostaining for WDR77 protein in the screening of patients with NS-FNMTC, and also whether this pathogenetic mechanism occurs in sporadic PTC.

### BRO1 Domain and CAAX Motif Containing (*BROX*)

The BRO1 domain and CAAX motif containing (*BROX*) gene, also known as *C1orf58*, has been associated with PTC from the analysis by WES of patients and non-affected relatives of five families with at least two family members affected by PTC (11). One family showed, in the three women with PTC, a new loss-of-function variant of *BROX* gene consisting of a frameshift deletion chr1: 222892283 (NM_001288579: c.119delG: p.Arg40fs) that was confirmed by Ssq and was absent from all internal and external databases. In another family, also with three sisters having PTC, a new *BROX* variant (chr1: 222886144 NM_144695: c.2898C>T) was detected in the two cases investigated. The patients had a mean age at diagnosis of 35.6 years (range 29-45 yrs.) with PTC in all cases including the classic types (one of them multifocal), the follicular variant and one papillary microcarcinoma (mPTC).

*BROX* gene encodes for a human protein that has a Bro1 domain-like sequence and a C-terminal thioester-linkage site of isoprenoid lipid. Bro1 domain is necessary for the morphogenesis of multivesicular bodies and is involved in the endosomal sorting of cargo proteins, including integrin and epidermal growth factor receptor (EGFR) degradation in lysosomes (56, 57). Considering that EGFR is a key regulator of cell growth and survival, Pasquali et al. (11) hypothesized that *BROX* haploinsufficiency alters EGFR degradation in follicular thyroid cells, with EGFR accumulation and aberrant cell growth. It remains unverified whether immunostaining for EGFR in PTC is useful for screening for NS-FNMTC associated with pathogenic germline variants of *BROX*.

### Protection of Telomeres 1 (*POT1*)

Protection of telomeres 1 (*POT1*), also known as *GLM9*, *CMM10*, and *HPOT1* encodes a nuclear protein involved in telomere maintenance. Patients with familial PTC display an imbalance of the telomere-telomerase complex in the peripheral blood, characterized by short telomeres and *hTERT* gene amplification (58). Relative telomere length (RTL) is also shorter in patients with FNMTC but is not associated with an altered copy number or expression in *hTERT*, *TRF1*, *TRF2*, *RAP1*, *TIN2*, *TPP1* and *POT1*/*TERF2IP* (59). In another study, the shorter telomeres observed in familial PTC are not linked to mutations or polymorphisms in the genes of the telomerase RNA component (*TERC*), *POT1* or *RAP1* either (60). Nevertheless *POT1* germline mutations have been implicated in susceptibility for melanoma, displastic nevi and FNMTC in some families (13, 14); and more recently, a novel germline variant (p.Val29Leu) of *POT1* gene was reported in one family affected solely by NS-FNMTC (16). In this study, the HEK293T cells carrying *POT1* p.Val29Leu showed increased telomere length in comparison to wild-type cells, supporting the hypothesis that this mutation (loss-of-function or reduced activity) causes telomere dysfunction and plays a role in predisposition to NS-FNMTC in this family (16). Patients with this novel *POT1* missense variant were diagnosed either with PTC (including mPTC), Hürthle (oncocytic) cell carcinoma (HCC), or benign thyroid nodules, however sometimes combining PTC and HCC, or coexisting with MNG (14, 16). The mean age of patients was 37.7 years (range 28-44 yrs.) and the female-to-male ratio was 3:1 (14, 16). Recent research using a functional variant approach has confirmed an association between a low frequency intronic regulatory *POT1* variant and subsequent thyroid malignancy in childhood cancer survivors (15). These results suggest that intronic variation in *POT1* may affect key protein-binding interactions that affect telomere maintenance and genomic integrity (15). Another recent study, however, including 4 Spanish families with familial PTC, did not detect potentially pathogenic germline mutations in *POT1* by WES, thus minimizing the possible role of this gene in NS-FNMTC (61).

### Forkhead Box E1 (FOXE1)

The forkhead box E1 (*FOXE1*) gene, formerly called *TTF2* (thyroid transcription factor 2), encodes a protein that functions as a transcription factor that plays a role in thyroid morphogenesis and migration. *FOXE1* is also important for thyroglobulin and thyroperoxidase gene expression, as well as for the maintenance of thyroid differentiation in adults (62, 63). By genome-wide association study (GWAS), Gudmundsson et al. (64) identified the SNP rs965513 (9q22.33) near the *FOXE1* gene, associated with an increased risk of PTC and FTC in the general population. *FOXE1* was also found associated with an increased risk of sporadic Japanese PTC (65). The genotyped study of a Spanish series of 615 cases and 525 controls also showed an association of the *FOXE1* gene with susceptibility to PTC and showed that the variant rs1867277 confers TC susceptibility through the recruitment of USF1/USF*2* transcription factors (66). Interestingly, rs1867277 was also reported to be in a strong link to the *FOXE1* region that encodes the polyalanine stretch of the protein in Caucasian patients with PTC (67). Subsequently, a high-throughput association study in a cohort of 238 families from France, Italy, and Greece with at least two members affected by PTC or FTC, confirmed the association between NS-FNMTC and the two SNPs rs965513 and rs10759944 located on chromosome 9p22.33 (17). SNPs rs965513 and rs1867277 and a polymorphic region determining the length of the *FOXE1* polyalanine (poly-Ala) tract were also associated with PTC in populations of Asian and European descent on univariate analysis, but the functional tests showed that variants rs965513 and rs1867277 independently contribute to genetic predisposition to PTC, while a contributing role of the *FOXE1* poly-Ala polymorphism could not be confirmed (68). Other GWAS in Spanish and Italian populations identified variations in the same 9q22 locus near *FOXE1* (rs7028661, rs7037324), associated with risk of PTC, including the follicular variant of PTC (≈18%) as well as FTC (≤13%) (18).

A study on sixty Portuguese families in which two or more first degree family members were affected with NS-FNMTC identified nine polymorphisms (rs1867277, rs7849497, rs1867278, rs1867280, rs965513, rs1867279, rs3021526, rs3021523, and poly-Ala) as well as one new variant (c.743C>G, p.Ala248Gly) of *FOXE1* (19). The authors demonstrated that the p.Ala248Gly variant promoted cell proliferation and migration, supporting that it may be involved in thyroid tumorigenesis. In the same study, TC patients who were carriers of the *FOXE1* p.Ala248Gly variant had PTCs (classic, follicular and tall cell variants), in some cases with additional somatic *BRAF* ^V600E^ mutations but not in *N*-, *K*- and *H-RAS* genes (19). Some researchers have shown that FOXE1 overexpression and translocation to the cytoplasm are phenotypic hallmarks of PTC cells (69). Furthermore, a higher nuclear FOXE1 immunohistochemical expression in tumor cells in the vicinity of the PTC border is associated with the presence of a risk allele of rs1867277 in the *FOXE1* 5 ‘untranslated region (UTR), as well as with pathological characteristics of PTC such as multifocality and capsular invasion (69). The practical utility of the immunohistochemical study of FOXE1 in diagnosis of thyroidectomy specimens either in the screening of familial PTC or as a prognostic marker needs confirmation.

### Hyaluronan Binding Protein 2 (*HABP2*)

Hyaluronan binding protein 2 (*HABP2*) gene encodes a member of the peptidase S1 family of serine proteases. It has been proposed that *HABP2* p.Gly534Glu variant is a susceptibility gene for NS-FNMTC in the USA population (20). This variant was associated with increased HABP2 protein immunoexpression in PTC samples from affected family members when compared to the normal adjacent thyroid tissue and samples from sporadic TC, which means that *HABP2* p.Gly534Glu variant would function as a dominant negative tumor-suppressor gene (20). In a subsequent study by Zhang et al. (21), the overall prevalence of *HABP2* p.Gly534Glu was six per 43 (14.0%) PTC patients from the 29 kindreds with NS-FNMTC and four per 29 (13.8%) kindreds; therefore, these results are apparently consistent with *HABP2* p.Gly534Glu being a susceptibility gene in a subgroup of FNMTC. Another different and non-pathogenic variant of *HABP2* has also been detected in PTCs (70). The consideration of *HABP2* p.Gly534Glu as a mutation, however, has been questioned given that due to the high population frequency of this variant, there is a high probability (>10%) that *HABP2* p.Gly534Glu will be present in four out of 29 families by chance; in addition, the probability of sharing the variant among affected relatives is also very high (12.5%), independent of the disease phenotype (71). In fact, the matching of the patients with controls from appropriate databases with a similar ancestry composition is essential (72).

In contrast to findings previously reported in the USA population (20), in a large series of 27 unrelated Italian families with FNMTC, Colombo et al. (73) found that: the p.Gly534Glu, rs7080536 genetic variant of *HABP2* did not segregate with TC, that non-affected people harbored this genetic variant, and that at an immunohistochemistry level, HABP2 was expressed in both tumor and matched control tissues, without differences between sporadic and familial cases. Finally, the *HABP2* variant was not found associated with PTC in Chinese (74), Caucasian American (75), Australian (76), British (77), Spanish (78), the Middle East (79), French (72), or Brazilian families with NS-NMTC (80). Neither was an association of the p.Gly534Glu variant of *HABP2* with sporadic PTC found (75, 79, 81, 82). In conclusion, it does not seem appropriate to consider a role of the p.Gly534Glu variant of *HABP2* in the pathogenesis of NS-FNMTC.

### SLIT-ROBO Rho GTPase Activating Protein 1 (*SRGAP1*)

SLIT-ROBO Rho GTPase activating protein 1 (*SRGAP1*), also known as *NMTC2* and *ARHGAP13*, encodes a protein that interacts with the transmembrane receptor ROBO1 to inactivate CDC42. SRGAP1 mediates multiple signaling pathways and participates in malignancies (83, 84). A genome-wide linkage analysis (GWLA) of 38 Caucasian non-Hispanic and Caucasian Hispanic families (the majority having 3 or more affected individuals with PTC) identified the *SRGAP1* as a candidate gene in PTC susceptibility (22). Two missense variants, p.Gln149His and p.Ala275Thr, localized in the Fes/CIP4 homology domain and another missense variant, p.Arg617Cys, located in the RhoGAP domain, were identified (22). In this study, biochemical assays demonstrated that the ability to inactivate CDC42 was severely impaired by the p.Gln149His and p.Arg617Cys variants (22). Although downregulation of SRGAP1 in colorectal cancer was associated with tumor progression and poor prognosis (84), no differences were detected in relation to the mean age at diagnosis and histological subtype of TC between families with and without 12q14 linkage (22). It remains to be determined whether the decrease in the immunohistochemical expression of SRGAP1 protein in colorectal cancer cells when compared to normal tissue detected by Feng Y, et al. (84), is also useful for the diagnosis and/or prognosis in cases of NS-FNMTC.

### Chromosome 14 Open Reading Frame 93 (*C14orf93/RTFC*)

Chromosome 14 open reading frame 93 (*C14orf93*) gene, also known as *RTFC* (regulator of thyroid function and cancer), regulates *in vitro* thyroid differentiation and *in vivo* thyroid function (85). *C14orf93* gene has also recently been identified as a susceptibility gene for NS-FNMTC in the Chinese population (23). The mutation p.Val205Met (c.613G>A) detected in the *C14orf93* gene of the patients with familial PTC was absent in the unaffected control individuals of matched geographical ancestry. Two additional p.Gly209Asp (c.626G>A) and p.Arg115Gln (c.344G>A) oncogenic mutations of *C14orf93* were identified in sporadic NMTC patients. In the same study, the oncogenic functions of p.Arg115Gln, p.Val205Met, and p.Gly209Asp *C14orf93* mutants were demonstrated by cell surviving assay, migration assay, and colony forming assays (23).

### NK2 Homeobox 1 (*NKX2-1*)

NK2 homeobox 1 (*NKX2-1*) gene, also known as *TTF1* (thyroid-specific transcription factor-1) and *BCH*, is located at 14q13.3. *NKX2-1* encodes a protein mainly involved in the transcription of thyroid-specific genes (*TG*, *TPO* and the *TSHR*), as well as in the development and maturation of the thyroid (86). A germline missense mutation (c.1016C>T) was identified in *TTF-1*/*NKX2.1* that led to a mutant TTF-1 protein (p.Ala339Val) in 4 of the 20 MNG/PTC patients (20%) after the targeted DNA sequencing of 20 PTC patients with a history of MNG, 284 PTC patients without a history of MNG and 349 healthy controls (24). The overexpression of p.Ala339Val *TTF-1* when transfected to rat normal thyroid cells was associated with increased cell proliferation including thyrotropin-independent growth, enhanced *STAT3* activation and impaired transcription of the thyroid-specific genes *TG*, *TSHR*, and *PAX8* (24). This p.Ala339Val mutation of *TTF1* was absent, however, in an Italian series of 63 patients with familial PTC (including classic forms, 15 cases of the follicular variant and one case of the warthin-like variant) as well as in unaffected family members (87).

Another association with TC risk reported by Gudmundsson et al. in 2009 (64), was for the risk allele T of the SNP rs944289, located at 14q13.3 near *NKX2-1* gene, although, rs944289 localizes 336 kb centromeric and downstream to *NKX2-1* (88). The SNP rs944289 was associated with an increased risk of sporadic PTC in the Japanese and Kazakh populations (65, 89). This SNP rs944289 was also found to predispose to PTC through a long intergenic noncoding RNA gene (lincRNA) named papillary thyroid carcinoma susceptibility candidate 3 (*PTCSC3*) located 3.2 kb downstream of rs944289 at 14q13.3 that has the characteristics of a tumor suppressor (47). Later, an increase in the frequency of the risk allele of rs944289 (T) in the members of a Chinese family with MNG, with and without PTC (bilateral and multicentric), has been reported (25). More recently, the association of the variant rs944289 with risk of both PTC and follicular thyroid adenoma in the Japanese population was confirmed, but neither *NKX2-1* nor *PTCSC3* could be confirmed as the risk genes for thyroid tumorigenesis (88).

### Dual Oxidase 2 (*DUOX2*)

Dual oxidase 2 (*DUOX2*) encodes a glycoprotein that is a member of the NADPH oxidase family. The DUOX2 enzyme generates hydrogen peroxide, a crucial electron acceptor for the thyroid peroxidase-catalyzed iodination and coupling reactions mediating thyroid hormone synthesis. DUOX2 mutations cause congenital hypothyroidism that may be phenotypically heterogeneous (90). Using WES, Bann DV, et al. (26) identified a novel p.Tyr1203His germline *DUOX2* mutation in a family that segregated as an autosomal dominant thyroid cancer phenotype. The proband presented with multifocal mPTC at 46 years of age and she had an extensive family history of TC and MNG. The authors detected a lower immunohistochemical expression of DUOX2 in a proband mPTC compared to a case of sporadic PTC, while another proband mPTC and normal thyroid tissue from both patients strongly stained for DUOX2, suggesting that p.Tyr1203His *DUOX2* heterozygosity does not dramatically reduce DUOX2 expression. In this same family, in addition to the germinal missense variant p.Tyr1203His (c.3607A>G) in DUOX2, the p.Gln61Arg somatic mutation in *KRAS* was detected in a follicular variant of mPTC and the somatic mutation of *BRAF* ^V600E^ in a classic form of mPTC (26). Unfortunately, the role of this germline variant could not be confirmed in a recent series of 33 unrelated Italian FNMTC kindreds (91).

### Mitogen-Activated Protein Kinase 5 (*MAP2K5*)

Mitogen-activated protein kinase 5 (*MAP2K5*), also known as *MEK5* and *MAPKK5*, encodes a dual specificity protein kinase that belongs to the MAP kinase family. After complete exome and target gene sequencing of 34 Chinese families with more than two first-degree relatives diagnosed as PTC without another familial syndrome, the recurrent genetic mutation of *MAP2K5* variants c.961G>A and c.1100T>C (p.Ala321Thr and p.Met367Thr) have been proposed as susceptibility loci for NS-FNMTC (27). Functional studies indicated that these two variants could consistently phosphorylate downstream protein ERK5 on site Ser731+Thr733 or Ser496, promoting nuclear translocation and subsequently altering target gene expressions. The same study revealed that *MAP2K5* variants p.Ala321Thr or p.Met367Thr can activate MAP2K5-ERK5 pathway, and subsequently induce malignancy (27). Consequently, while the classic MEK1/2 –ERK1/2 activity is essential in the tumorigenesis of sporadic NMTC, its alternative pathway, MAP2K5 – ERK5 activation, is likely responsible for a subgroup of NS-FNMTC cases. The absence of these *MAP2K5* germline variants in a series of 33 unrelated NS-FNMTC Italian families (92) has been attributed to different sensitivity of the techniques used (WES versus Ssq) and/or ethnic differences (93).

### Serine/Arginine Repetitive Matrix 2 (*SRRM2*)

Serine/arginine repetitive matrix 2 (*SRRM2*) gene is also known as *CWF21*, *Cwc21*, *300-KD*, *SRL300*, *SRm300* and *HSPC075*. By combining genotyping, haplotype analysis, WES and genetic linkage analysis, a germline mutation in *SRRM2* was implicated in PTC predisposition (28). The heterozygous variant c.1037C>T (p.Ser346Phe; rs149019598) cosegregated with PTC in one family displaying Mendelian-like inheritance in two generations. All cases of TC in this family were PTC, including three cases of mPTC (two classic PTC and one follicular variant of PTC) with three of the affected individuals ≤ 25 years of age (28). The researchers postulated that the p.Ser346Phe mutation in *SRRM2*, a splicing factor gene, predisposes to PTC by affecting alternative splicing of unidentified downstream target genes (28).

### NADH : Ubiquinone Oxidoreductase Subunit A13 (*NDUFA13*)

NADH:ubiquinone oxidoreductase subunit A13 (*NDUFA13*) gene, also known as *GRIM19*, is located at 19p13.11. *NDUFA13* as well as the *TIMM44* and *MYO1F* genes have been associated to the oncocytic phenotype and the *TCO* (thyroid tumors with cell oxyphilia) locus at 19q13.2 (reviewed by Correia M et al. (49). *NDUFA13* encodes a protein that exerts a dual function: (i) it is essential for assembly and function of the complex I of the mitochondrial respiratory chain, and (ii) it induces apoptosis in a number of cell lines upon treatment with interferon-beta and retinoic acid (94). Somatic and germline mutations in *NDUFA13* have been linked to benign and malignant mitochondrion-rich (Hürthle cell/oncocytic) tumors of the thyroid (29).

### Translocase of Inner Mitochondrial Membrane 44 (*TIMM44*)

Translocase of inner mitochondrial membrane 44 (*TIMM44*) gene, also known as *TIM44*, is located in the *TCO* region at 19q13.2 (see chromosomal loci section below). *TIMM44* encodes a peripheral membrane protein associated with the mitochondrial inner membrane translocase, implied in the import of proteins across the mitochondrial inner membrane and into the mitochondrial matrix. This protein also participates in the union of mitochondrial heat shock protein 70 (mtHsp70) to the translocase of inner mitochondrial membrane 23 (TIM23) complex (95). The systematic screening of 14 candidate genes mapping to the region of linkage in eight Caucasian families with benign and malignant oncocytic thyroid tumors, led to the identification of two novel variants respectively in exon 9 and exon 13 of *TIMM44*, which co-segregated with the *TCO* phenotype (30). Although the *in vitro* functional studies did not show a dramatic loss of function effects for the mutant alleles, the authors postulated that the subtle effects detected might still alter *TIMM44* function and thus promote oncocytic tumor development (30). Interestingly, a germline heterozygous mutation in the *KEAP1* gene, also located at 19p13.2-q12, was found in a Japanese MNG family (96).

### NOP53 Ribosome Biogenesis Factor (*NOP53*)

NOP53 ribosome biogenesis factor (*NOP53*) gene, also known as *PICT1*, *PICT-1* and *GLTSCR2*, encodes a nucleolar protein that participates in ribosoma biogenesis. *NOP53* regulates the activation of p53/TP53 in response to ribosome biogenesis perturbation, DNA damage and other conditions (97). *NOP53* also participates in the autophagy, maintenance of chromosomal stability and mitotic integrity during nuclear division, suggesting that *NOP53* expression may be a critical event in malignancy (98). The germline c.91G>C, p.Asp31His variant (dbSNP: rs78530808) in *NOP53* gene was identified in all affected members in three Spanish families with two or more members with NS-FNMTC (31). The patients with TC (10 PTC and 1 HCC) were 7 women and 4 men with a mean age at diagnosis of 40.7 years (range 24-57 yrs.); concurrent MNG and toxic MNG occurred in 2 and 2 cases respectively. In this study, functional data in cell lines were consistent with an oncogenic role for *NOP53*. Interestingly, the thyroid tumor tissue from the affected patients showed higher immunohistochemical expression of NOP53 (intense staining), compared to adjacent normal thyroid tissue (31). Therefore, given the relatively high frequency of the variant in the general population, *NOP53* is likely a low-penetrant gene involved in NS-FNMTC, possibly a modifier (31). 

### Phospholipase C Beta 1 (*PLCB1*)

Phospholipase C beta 1 (*PLCB1*) gene is also known as *DEE12*, *PLC-I*, *EIEE12*, *PLC154*, *PLC-154* and *PLC-beta-1*. *PLCB1* encodes a protein that catalyzes the formation of inositol 1,4,5-trisphosphate and diacylglycerol from phosphatidylinositol 4,5-bisphosphate. This reaction uses calcium as a cofactor and is important in the intracellular transduction of many extracellular signals. The PLCB1 protein is required for polarization of the human embryo, participates in the regulation of follicular cell growth, and is frequently upregulated in human cancer activating PI3K/AKT signaling axis (99, 100). An intronic InDel (≈1000 bp) in the *PLCB1* gene was linked to a familiar form of euthyroid MNG of adolescent onset (7 female and 2 male), in which some family members developed PTC (32, 33). This intronic InDel may function through overexpression, and increased PLC activity has been reported in thyroid neoplasms. Due to the existence of a binding site for the estrogen receptor alpha within the deletion, the authors have suggested a possible relationship with the higher prevalence of thyroid diseases in women and the higher incidence of thyroid disorders immediately following puberty (33). Therefore, and based on functional studies, the authors have also suggested that the InDel may contribute to MNG development through overexpression of PLCB1 (33).

The pathological characteristics of the lesions described in the thyroidectomy samples were identical and therefore could serve to recognize this condition associated with NS-FNMTC (32, 34). The thyroidectomy specimens showed multiple nodules enlarging an otherwise normal tissue. Microscopically, the nodules were typically well demarcated from the normal thyroid and showed colloid-rich follicles as well as frequent micropapillary projections lined by epithelial cells with small round nuclei (papilloid adenomas) (32, 34). This peculiar form of MNG associated with *PLCB1* is clearly different from the common MNG where the ill-defined nodules lack the micropapillary projections, and the background thyroid also shows similar but less marked changes (34). The morphology of PTCs (including the follicular variant) in this setting, however, is not different from sporadic PTC (32). It remains unconfirmed whether the overexpression of PLCB1 may provide a biomarker to identify MNG patients more prone to progress to PTC.

### Checkpoint Kinase 2 (*CHEK2*)

Checkpoint kinase 2 (*CHEK2*) gene is also known as *CDS1*, *CHK2*, *LFS2*, *RAD53* and *hCds1*. The protein encoded by *CHEK2* is a cell cycle checkpoint regulator and putative tumor suppressor. When activated, this protein is known to inhibit CDC25C phosphatase, preventing entry into mitosis, and has been shown to stabilize the tumor suppressor protein p53, leading to cell cycle arrest in G1 (101). In addition, CHEK2 protein interacts with and phosphorylates BRCA1, allowing BRCA1 to restore survival after DNA damage (101). *CHEK2* germline mutations impair the DNA damage repair process and have been associated with hereditary cancer in various organs such as breast, colon, kidney and thyroid (102).

A multiple cancer study in Polish patients identified an increased risk of PTC, particularly in carriers of *CHEK2* truncations (103). A subsequent study in the same population confirmed that a *CHEK2* truncating mutation (1100delC, IVS2+1G>A, del5395) was associated with odds ratio of 5.7 (p= 0.006), and a *CHEK2* missense p.Ile157Thr mutation was associated with odds ratio of 2.8 (p= 0.0001) for PTC (104). In a more recent Polish series that included 1547 unselected PTC patients (1358 women and 189 men), *CHEK2* mutations were found in 240 (15.5%) patients (37). A *CHEK2* p.Ile157Thr missense mutation was found in 12.3%, and *CHEK2* truncating mutations (IVS2+1G>A, del5395, 1100delC) were found in 2.8%. While truncating mutations were more common in women and were associated with vascular invasion and intermediate or high initial risk in multivariate analysis, no significant differences were observed in patients with the p.Ile157Thr missense mutation (37).

Siolek M, et al. (104) evidenced a *CHEK2* mutation in 63% of 11 women with thyroid and breast cancer. Using WGS, Wang Y, et al. (105), identified the rs555607708 (CHEK2*1100delC) variant among 17 families with three or more PTC patients showing a Mendelian-like mode of inheritance. In these families with PTC and MNG (one of them also with melanoma), the ratio female to male in PTC patients was 76:29 (105). The CHEK2*1100delC variant is a well-established breast cancer risk variant in European populations (106) and was also associated with prostate cancer (107). A more recent study, reported a germline *CHEK2* pathogenic variant (c.596dupA, p.Tyr199Ter) in the proband and in five additional members of a Portuguese Roma family with MNG and/or PTC; in this same family, two women were affected by breast cancer and classic PTC and one of the men by prostate cancer and PTC (38). A novel heterozygous germline mutation in *CHEK2* (c.417C>A) was detected in all available affected members of a kindred with familial PTC (35). This *CHEK2* c.417C>A variant introduces a premature termination codon (p.Tyr199Ter) and was associated with a lower immunohistochemical expression of the CHEK2 protein compared to sporadic cases without the variant (35). The p.Tyr139Xaa loss-of-function variant also led to reduced p53 phosphorylation and decreased p53 protein level (35). Two rare missense variants (p.Arg180Cys and p.His371Tyr) in *CHEK2* were also identified in 5 (2%) of 242 patients with sporadic PTC in the same study (35).

Both somatic *BRAF* ^V600E^ and germline *CHEK2* mutations coexisted in 10.8% of a series of 427 unselected PTC patients (377 women and 50 men) treated in single center of Poland (108). Although *CHEK2* mutations were significantly associated with advanced age, coexistence with *BRAF* ^V600E^ mutations was not associated with more aggressive clinicopathologic features of PTC, poorer response to treatment, or poorer outcome of the disease (108). A more recent WGS identified three potentially disease-causing germline variants in *CHEK2* (p.Glu239Lys), *TIAM1* (p.Arg1053Cys), and *EWSR1* (p.Ala327Asp) in a family with aggregation of PTC (including micro-PTC), insular thyroid carcinoma and benign thyroid nodules (109). Due to the fact that all three genes are part of cell cycle regulation and DNA damage repair pathways, the authors have suggested a polygenic mode of inheritance of TC in this family (109).

According to all these data, when a germline *CHEK2* mutation is detected, the screening for TC and other tumors should be carried out together with the genetic screening of the family members (36, 105, 109, 110). The diagnostic utility of the lower immunohistochemical expression of the CHEK2 protein (and p53) in thyroid tumors from patients with a germline *CHEK2* mutation is pending confirmation.

### Other Genes

Pathogenic/likely pathogenic sequence variants in seven novel genes were detected (p.Asp283Asn *ANXA3*, p.Tyr157Ser *NTN4*, p.Gly172Trp *SERPINA1*, p.Gly188Ser *FKBP10*, p.Arg937Cys *PLEKHG5*, p.Leu32Gln *P2RX5*, and p.Gln76Ter *SAPCD1*) by WES in three Brazilian families with hereditary PTC (12). The eleven patients (8 women and 3 men), with a mean age of 41.5 years (range 18 to 70 yrs.), have classic or follicular variants of PTC, except in one case with HCC (12). Decreased expression of *ANXA3* in PTC at both the protein (annexin A3) and mRNA levels correlates with tumor progression in sporadic PTC (111). *NTN4*/*PRO3091* gene encodes a member of the netrin family of proteins (netrin 4), which participates in various biological processes, including tumorogenesis and angiogenesis; in fact, netrin-4 is reduced in breast cancer tissue and is associated with breast cancer cell migration and invasion (112). *SERPINA1* gene encodes a serine protease inhibitor (serpin family A member 1) differentially expressed in sporadic PTC (113–115). SERPINA1 (alpha1AT) expression was also significantly associated with radiation carcinogenesis (I-131 exposure) in tumor tissues from post-Chernobyl PTC cases (116). The protein encoded by *FKBP10* gene belongs to the FKBP-type peptidyl-prolyl cis/trans isomerase family and is involved in cell metabolism and autophagy (117); it has also been implicated in the development of cancers in various organs (117–119). The *PLEKHG5* (pleckstrin homology and RhoGEF domain containing G5) gene encodes a protein that activates the nuclear factor kappa B (NFKB1) signaling pathway and is a novel prognostic biomarker in glioma patients (120, 121). *P2RX5* (purinergic receptor P2X 5), also known as P2X5/*LRH-1* encodes a receptor for adenosine 5’-triphosphate (ATP) and adenosine that functions as a ligand-gated ion channel; this receptor can be aberrantly expressed in a subset of primary solid tumor specimens derived from renal cell carcinoma, brain cancer, colon and breast cancer patients (122, 123). The suppressor APC domain containing 1 (*SAPCD1*) gene is involved in DNA repair and is underexpressed in breast cancer tissue (124). The *SAPCD1* variant (p.Gln76Ter) reported by Sarquis et al. in a family with PTC has also been described as associated with a risk of lung cancer (125). All these variants reported by Sarquis et al. (12), however, require validation in other families with NS-FNMTC.

By GWAS, a strong association between the single-nucleotide polymorphism (SNP) rs2439302 with the risk of nonmedullary TC and the expression of *NRG1* gene has been reported by Gudmundsson et al. (126). *NRG1* (neuregulin 1), is located at 8p12, and encodes a membrane glycoprotein that mediates cell-cell signaling and plays a role in development of multiple organ systems as well as in several malignancies (127). An association between *NRG1* rs2439302 and PTC was confirmed in another study that additionally showed a relationship with tumor multifocality and lymph node metastases (128). A more recent GWAS in Koreans also showed that the variants of *NRG1* are associated with lymph node metastases, especially in *BRAF* ^V600^-mutated PTC (128).

An association between TC and SNP rs966423, located in the *DIRC3* (disrupted in renal carcinoma 3) gene mapping to the 2q35 locus was identified by GWAS in the Icelandic population (126). This association was confirmed by the same group for the SNP rs11693806, mapping in the *DIRC3* gene (129) and also in Asian population (130). It has been postulated that changes in *DIRC3* would alter TSH levels and secondarily promote development of TC as a consequence of less differentiation of the thyroid follicular epithelium (126, 131).

## Chromosomal Loci Associated With NS-FNMTC

Familial papillary thyroid carcinoma with papillary renal neoplasia (*fPTC*/*PRN*) gene was mapped to 1q21 region in an American family with PTC (and benign thyroid nodules) as well as benign and malignant papillary renal neoplasms (39). A genome-wide SNP array-based linkage analysis of American and Italian families with 2 or more first degree relatives with well-differentiated thyroid cancer of follicular-cell origin (PTC, FTC and HCC), identified the same chromosomal locus 1q21 along with locus 6q21 associated with the risk of TC (40).

Non-medullary thyroid carcinoma 3 (NMTC3) gene was identified in 2q21 as a susceptibility gene in a large Tasmanian family with PTC (classic and follicular variant), and benign thyroid nodules (FTA and MNG) (41). Another subsequent linkage analysis in 10 FNMTC families, nine of which presented with cell oxyphilia, confirmed the existence of susceptibility genes for NS-FNMTC at 2q21 as well as at 19p13 (42). It has also been suggested that the interaction of these two loci could significantly increase the risk of TC (42). The occurrence of loss of heterozygosity (LOH) at the 19p13.2 (*TCO*) and 2q21 (*NMTC1*) loci in tumors from familial clusters of NS-FNMTC (with and without cell oxyphilia), supports that the inactivation of putative genes in these regions, acting as tumor-suppressors, may be involved in the development of TC in this setting (43).

A susceptibility locus 8p23.1-p22 also known as *FTEN* (familial thyroid epithelial neoplasia) has been reported in a Portuguese family with 11 members affected with benign thyroid lesions (MNG or FTA) and five affected with PTC (44).

Papillary thyroid carcinoma susceptibility candidate 1 (*PTCSC1*) in locus 8q24 has been identified, by GWLA, in a family with PTC, benign thyroid nodules and melanoma, as a predisposing locus for PTC (45). More recent studies have also identified variants in this 8q24 locus associated with risk of prostate cancer, colorectal cancer, breast cancer, bladder cancer, stomach cancer, glioma and thyroid cancer (132–134).

In a European GWAS involving 1820 cases and 2410 controls, rare alleles of three SNPs (rs2997312, rs10788123 and rs1254167) at 10q26.12 in intronic regions of *WDR11-DT* (WDR11 divergent transcript) evidenced an association with an important risk of TC (18). *WDR11-DT*/*WDR11-AS1* was associated with survival in glioblastoma (135) and it has been proposed that disruption of the WDR11 regulation axis could represent a pathogenic mechanism of TC (18). Interestingly, in the same European study, the rare allele of rs4075570 at 6q14.1 conferred protection (18).

Multinodular goiter 1A (*MNG1*) in a locus on chromosome 14q has been identified in a single Canadian family with 18 cases of adolescent onset nontoxic MNG in which a few members also had PTC (46). Curiously, a GWAS of PTC identified a unique, long, intergenic, noncoding RNA gene (lincRNA) named papillary thyroid carcinoma susceptibility candidate 3 (*PTCSC3*), with the characteristics of a tumor suppressor, located 3.2 kb downstream of rs944289 at 14q.13.3 (47). It is probable that some of these cases reported in children with MNG and TC could really be secondary to a germline mutation of the *DICER1* gene located at 14q32.13 (50–52), or could be due to germinal mutations of the *NKX2-1* gene located at 14q13.3 (25).

Thyroid tumor with cell oxyphilia (*TCO*/*TCO1*) was mapped to chromosome 19p13.2 in a French pedigree with benign and malignant tumors showing variable eosinophilia, including oxyphilic PTC and HCC (48). The *TIMM44* and *MYO1F* genes at 19p13.2 and the *NDUFA13*/*GRIM19* gene at 19p13.11 are related to the oncocytic phenoptype (see above).

MicroRNAs (miRNAs or miRs) are small single-stranded non-coding RNAs that regulate gene expression by degrading mRNAs or suppressing translation. miRNAs play an important role in many biological and metabolic pathways, including thyroid carcinoma (136). By genome-wide mRNA expression microarray both miR-20a (13q31.3) and miR-886-3p (5q31.2) have been identified as susceptibility loci in kindreds with NS-FNMTC; in the same study, functional data using thyroid cancer lines supported the role of these two miRNAs in the initiation and/or progression of PTC (137).

## Conclusions

Recent research shows that the genetic basis of NS-FNMTC is complex and heterogeneous, involving not only a monogenic, but also a polygenic mode of inheritance in some families. Most cases of NS-FNMTC are PTC and predominantly in women, the same as occurs with its sporadic counterpart. Both familial cases of PTC (classic and follicular variant) as well as other types of well-differentiated carcinoma of the follicular lineage are usually accompanied by benign thyroid nodules (FTA and/or MNG). While several genes have been proposed as being involved in NS-FNMTC, in familial tumors with an oncocytic (Hürthle cell) phenotype, *TCO*, *NDUFA13*/*GRIM19*, *TIMM44* and *MYO1F* have been identified as susceptibility genes. Interestingly, germline mutations in non-syndromic familial PTC impact multiple processes (proliferation, migration and/or cell survival, telomere dysfunction, tumor progression, autophagy, mitochondrial metabolism, chromosomal stability, angiogenesis, etc.), while somatic events are grouped mainly within the mitogen-activated protein kinase (MAPK) signaling pathway. Downregulation, overexpression or translocation of various proteins secondary to genetic alterations have been reported in some tumors of NS-FNMTC cases; the usefulness of these findings in diagnosis, however, still requires further confirmation. Given that NS-FNMTC and sporadic non-medullary TC share the same morphology and somatic mutations, the same targeted therapies could be used at present, if necessary, until more specific targeted treatments become available.

## Author Contributions

All authors contributed to conception of the manuscript. MS-A, SC-G, IA-N, and JC-T did literature research and wrote the first draft of the manuscript. GR-C, CB-S, and JP-R critically revised the manuscript. All authors contributed to manuscript revision, read, and approved the submitted version.

## Funding

This work was supported by grant n° PI19/01316 from Instituto de Salud Carlos III, State Research Agency and Ministry of Science and Innovation (Spain), with the participation of European FEDER funds.

## Conflict of Interest

The authors declare that the research was conducted in the absence of any commercial or financial relationships that could be construed as a potential conflict of interest.

## Publisher’s Note

All claims expressed in this article are solely those of the authors and do not necessarily represent those of their affiliated organizations, or those of the publisher, the editors and the reviewers. Any product that may be evaluated in this article, or claim that may be made by its manufacturer, is not guaranteed or endorsed by the publisher.
